# Rice husk ash addition to acid red soil improves the soil property and cotton seedling growth

**DOI:** 10.1038/s41598-022-05199-7

**Published:** 2022-02-01

**Authors:** Mengyao Yin, Xuan Li, Qian Liu, Feiyu Tang

**Affiliations:** Key Laboratory of Crop Physiology, Ecology and Genetic Breeding, College of Agronomy, Ministry of Education, Jiangxi Agricultural University, Nanchang, 330045 China

**Keywords:** Plant sciences, Environmental sciences, Solid Earth sciences

## Abstract

Red soil is characterized by poor physico-chemical properties and low nutrient availability. The present study aimed to examine rich husk ash (RHA) incorporation into red soil at various rates effects on its properties and the growth of cotton seedlings under a plug-seeding in tray experiment. Bulk density was decreased, and water holding capacity and total porosity were increased in red soil with increasing application rate of RHA. The addition of RHA counteracts the acidity of red soil and improves the nutrient availability to plants. The RHA incorporated soils favored the growth of cotton seedlings with improved shoot morphological traits and root architectures. The application rate at a volume ratio of 1:1 of RHA to red soil was found to be optimal for growing cotton seedlings in the present study. The mixture of RHA and red soil at a 1:1 volume ratio plus 2 g L^−1^ super absorbent polymers exhibited a high nursing seedling efficiency comparable to a commercial growing media under the condition of foliar application of mepiquat chloride at the one-true-leaf stage. RHA can be a promising substitute for peat as growing media for nursing cotton seedlings.

## Introduction

There are around 10.8 Mha of red soil in Jiangxi province China representing 64% of the total land area^1^. Red soil is derived from Quatenary red clay and categorized as Ferralic Cambisol^2,3^, which is characterized by poor chemical and physical characteristics such as low pH value and cation exchange capacity (CEC), low fertility (scarcity of potassium K, phosphorus P and calcium Ca, etc.), and high content of activated aluminum ion (Al^3+^), bad aeration and thin organic soil layer^1,3^. Cotton is grown widely in the hill red soil region of the middle-north section of Jiangxi province as a staple industrial crop^4^. It is difficult for the type of soil to produce comparatively high cotton lint yield due to its intrinsic drawback^5^. Thus, it is imperative to ameliorate the physical, chemical and biological properties of red soil for further enhancement of cotton productivity.

Jiangxi province is known as the largest third paddy rice production region in China with an annual production area of 3.30 Mha and a total yield above 20 million tons over the last decade^6^. Rice husk is a main byproduct during the milling process of paddy rice accounting for about 20–22% of rice kernel by weight^7,8^. The disposition of rich husk remains a main issue for the rice mill industry. Inappropriate disposition methods such as onsite burning, open dumping or land-filling may bring serious threats to environmental sustainability^8^. Rice husk can be used as fuel to generate electricity on a commercial scale^9^. The residue ash is called rice husk ash (RHA) after the combustion of rice husk. Depending on whether the combustion is complete or incomplete, rice husk ash is classified as white (WRHA) and black (BRHA) ones, or RHA1 and RHA2 with RHA1 being longer exposed to furnace than RHA2^10^. At present, RHA is mainly utilized as a pozzolanic material, adsorbent and source of silica, etc.^11^, but its usage in the agriculture industry is still scarce.

RHA is rich in chemical elements like K, P, Ca, Magnesium (Mg) and silicon (Si), and moderately alkaline and has considerable neutralizing power which confers it an application of fertilizer^10,12^. RHA contains three-layered structures: internal, external and interface with interstitial and honeycombed pores^11^. Scanning electron micrograph (SEM) photograph of RHA indicated its high porosity leading to a huge specific surface area (SSA) and superior absorptive ability^13^. Similarly, Singh et al. (2019) also observed the presence of various macro- and micro-pores on the surface of RHA^14^. Thus, RHA can be employed to remediate the heavy metal contaminated soil. The incorporation of RHA to Pb contaminated soil mitigated Pb damage to *R. communis*, and enhanced the uptake of nutrients and the activities of antioxidant enzymes^15^. RHA significantly reduced inorganic arsenic (As) accumulation in rice grains^16^. In addition, RHA has been widely used as an absorbent for the removal of lead (Pb(II) and mercury (Hg(II)) from an aqueous solution^11^. In comparison to its role as a soil amendment, the utilization of RHA as growing media has drawn much less attention^17^. Virtually, RHA is a potential substitute for peat in growing media due to its chemical and physical attributes similar to biochar which is deemed as a promising replacement for peat^10,17^. Biochar and peat shared the common physico-chemical properties such as high water and air holding ability and CEC and lightweight^18–20^, but peat is a non-renewable resource and its regeneration takes a very long time^21^. Couples of types of biochars usage as a growing media were investigated and characterized by improved soil properties and seedlings growth^21,22^. Nevertheless, RHA does not be examined for its potential application value as a growing media.

Jiangxi province is located in subtropical monsoon climate region with excessive precipitation and frequent hit of cold wave from the north at the cotton seedling stage. Direct seeding is difficult to ensure full standing of cotton seedlings. Thus, seedling transplantation technology is widely adopted by cotton growers in that production area. Commercial growing media is usually not cheap specifically the transport cost is very high, and in turn increases the financial pressure on farmers. The manufacture of rice husk biochar (RHB) commonly takes a longer time and consumes considerable electricity, and therefore is less profitable economically. By contrast, RHA is abundantly available at a throw-away price as a byproduct of rice milling and steam and electricity generations. Certain properties of RHA above-mentioned may offset the inherent shortage of acid red soil. In the present study, we hypothesized that RHA incorporation into acid red soil can ameliorate the physico-chemical properties of soil and improve the growth parameters of cotton seedlings. The objectives of this study were to (i) examine the effect of RHA application as a growing media substrate on the physical and chemical characteristics of red soil; (ii) to determine the influence of RHA application on the physiological and growth parameters of cotton seedlings; (iii) verify whether there are significant differences or not in nursing seedlings between RHA media and a widely used commercial growing media. If RHA is utilized as a growing media, it can not only avoid the damage to the environment but also bring an economic benefit.

## Materials and methods

### Rice husk ash, red soil and treatment details

RHA was taken from a local rice mill in which rice husk was burnt to produce steam for the processing of paddy rice. The RHA shares multiple common physical and chemical characteristics with rice husk biochar (RHB) which is the product of the slow pyrolysis of rice husk under little or no oxygen condition^10,15,16^. Compared to RHA, RHB had greater carbon content, nitrogen content, neutralizing power, pH value, CEC, and SSA, and much less silicon content, but no difference in macro-and micro nutrients including K, P, Ca, Mg, Manganese (Mn), Iron (Fe)^10,12^. In addition, the manufacture of RHB typically consumes substantial energy and takes longer time relative to RHA as a by-product of the mill industry. The RHA was water-saturated and black-colored when applied to soil. Red soil was taken from an uncultivated soil layer at a depth of 30 cm in the campus of Jiangxi Agricultural University, Nanchang China (28° 46′ N, 115° 55′ E), which was classified as Ferralic Cambisol^2,3^. The usage of uncultivated soil aimed to minimize herbicide, pesticide and pathogen residuals influence on cotton seedlings. The soil was air-dried, and then ground finely to pass through a sieve of 2 mm mesh. RHA was added to the soil one week before seeding at volume ratios of 1:0, 1:1, 2:1, and 1:2 (soil: RHA), and then mixed thoroughly by hand. The mixtures of soil versus RHA at volume ratios of 1:0, 1:1, 2:1, and 1:2 were defined as T0 (pure soil, the control), T1, T2, T3, respectively in experiment 1.

A second experiment was conducted to assess the effects of an optimized synthetic growing media formula (red soil combined with RHA at a ratio of 1:1 v/v, 2 g L^−1^ super absorbent polymers (SAP) (chemical name: potassium polyacrylamide, Renqiu Chemical Industry Co. Ltd, Renqiu city China) on cotton seedling growth. The formula was evidenced to perform best in previous preliminary tests. Experiment 2 included the following four treatments: T0 (pure soil), T5 (pure soil + 50 mg L^−1^ mepiquat chloride (MC) foliar painting at the one-true-leaf stage), T6 (red soil combined with RHA at a ratio of 1:1 v/v + 2 g L^−1^ SAP + 50 mg L^−1^ MC foliar painting at the one-true-leaf stage), and T7 (commercial nutrient soil with a trademark name of HUANUO, Shenyang Haoyuan Environmental Service Co. Ltd, Shenyang city China, plus 50 mg L^−1^ MC foliar painting at the one-true-leaf stage). The commercial nutrient soil contains peat, perlite, straw fibers, carbendazim, and rooting power, etc., as shown in the operation instruction.

### Experimental design and growth conditions

Cotton seeds were planted in 72-cave plastic trays with 37 cm in length and 28 cm in width. The upper and bottom diameters of the cave were 3.5 cm and 1 cm, respectively, and the total volume of each cave was 25 mL. The two experiments were arranged in a randomized complete block design with four treatments, and each treatment was repeated triple. Each of the plastic trays represented a replication and seventy-two seeds in each. The growth conditions in the greenhouse were set to be as follows: temperature 28/25 °C (day/night), photoperiod 16/8 h (light/dark), and light intensity 160 μmol m^−2^ s^−1^.

### Measurements

Ten normal seedlings were randomly sampled from each of plastic trays for measurement of the physiological and growth parameters at the three-true-leaf stage (about 1 month after seeding later), and then separated into three parts: root, stem, and leaf. A portion of subsamples was oven-dried at 105 °C for 30 min and then at 70 °C to constant weight. The fresh and dry weights were recorded, respectively. Dry subsamples were ground into fine powders for the determination of hexose, sucrose, starch, total carbon, and total nitrogen. The growing media with various ratios of red soil to RHA was subjected to analysis of physico-chemical properties at the end of the experiment. Soil bulk density (BD) was determined by the cutting-ring method as described by Liu^23^. Total porosity and water holding capacity (WHC) were measured according to Chang et al.^22^. pH value was measured in a 1:2.5 soil to deionized water mixture^12^. Available phosphorus was determined by the Olsen-P method, and Available potassium was extracted by ammonium acetate solution and quantified by a flame photometer^24^. Organic matter was measured following the titanium trichloride—potassium dichromate method^24^. Soil total nitrogen quantification was done by the Kjeldahl method. Nitrate nitrogen and ammoniacal nitrogen were determined according to the phenol-disulphonic acid and indophenol blue colorimetric methods, respectively^24^. Emergence rate was calculated as the percentage of survival seedlings to total seeds for each of treatments at the two cotyledons unfolding stage. Plant height was defined as the length from the base to the apex of plant. Root to shoot ratio was termed as the ratio of root dry weight to shoot dry weight. Stem diameter was measured at the middle of the hypocotyl using a vernier caliper scale. Seed index (SI) and G value were calculated in terms of the following functions^25^.

1 $$ SI = \left( {\frac{SD}{{PH}} + \frac{RDW}{{SDW}}} \right)  \times \left( {RDW + SDW} \right), $$

2 $$ G \,value = \frac{(RDW + SDW)}{{growth \,period}}, $$

where *SI* represents seed index; *SD*: stem diameter; *PH*: plant height; *RDW*: root dry weight; *SDW*: shoot dry weight; *growth period* denotes days during seeding to sampling.

Roots were rinsed free of soil with tap water, and scanned by a root scanner (Expression 12000XL, J331B, Japan). Root architecture parameters were recorded by WinRHIZO software (WinRHIZO Pro, Regent Instruments, QC, Canada) including total root length, root surface area, root volume, average root diameter, and the number of root tips. The exaction and determination of nonstructural carbohydrates (hexose, sucrose, and starch) were conducted following our previous protocols^26,27^. Total carbon in seedling organs was determined using Multi N/C 2100 analyzer with HT 1300 solid module (Analytik-Jena, Germany) and total nitrogen using the Kjeldahl method. Root vigor was measured using the 2,3,5-triphenyl tetrazolium chloride (TTC) method as described by Chen^28^.

### Statistical analysis

All data were subjected to one-way Analysis of Variance (ANOVA) and means were separated by Duncan’s Multiple Range Test at a probability of 0.05 level using the SPSS package (ver. 18.0). The figures were produced by Origin 8.5. All methods and collection of plant materials were performed in accordance with the relevant institutional, national, and international guidelines and legislation.

## Results

### Properties of red soil, RHA and their mixtures

The chemical properties of red soil and RHA were listed in Table 1. Soil pH, available P and K, organic matter, and total N were found higher for RHA as compared to the red soil. As expected, red soil is acidic and RHA is moderately alkaline. RHA incorporation to red soil ameliorated its physical properties (Table 2). Compared to T0 (pure red soil), T1 (1:1 of red soil to RHA) and T3 (1:2 of red soil to RHA) decreased bulk density and increased total porosity. WHC was increased as the rate of RHA application was enhanced. RHA application also improved the chemical characteristics of red soil (Table 3). Diverse ratios of the mixture of RHA and red soil had greater pH value, available P and K, organic matter and total N relative to pure red soil (T0). The more the addition of RHA to red soil, the greater these chemical characteristics in the mixtures. Ammonium nitrogen and nitrate nitrogen in red soil were enhanced slightly by RHA application. Taken together, RHA addition to red soil improved both physical and chemical properties. Bulk density was decreased by 4.67% to 58.88% while total porosity, available P, available K and organic matter were increased by 5.04 to 55.47%, 2.2 to 9.6 folds, 1.2 to 3.1 folds and 2.4 to 7.6 folds, respectively.

**Table 1:** Chemical properties of rice husk ash (RHA) and red soil**.** Values followed by different letters within the same column are statistically different at P ≤ 0.05 level. Values are presented as means ± SD.The same as below.

Treatment	pH value	Available P (mg kg^−1^)	Available K (mg kg^−1^)	Organic matter (g kg^−1^)	Total N (%)
Red soil	4.46 ± 0.20b	5.85 ± 0.64b	100.00 ± 0.00b	6.85 ± 0.35b	0.04 ± 0.00b
RHA	7.89 ± 0.08a	78.70 ± 0.28a	833.50 ± 9.19a	390.20 ± 15.56a	0.09 ± 0.00a

**Table 2:** Physical properties of the mixtures of rice husk ash and red soil with various.

Growing media	Bulk density (g cm^−3^)	Water holding capacity (%)	Total porosity (%)
T0	1.07 ± 0.04a	56.30 ± 1.18d	51.43 ± 1.88c
T1	0.72 ± 0.04b	93.77 ± 2.25b	67.30 ± 1.65b
T2	1.02 ± 0.01a	80.02 ± 1.87c	54.02 ± 0.30c
T3	0.44 ± 0.05c	132.13 ± 10.66a	79.96 ± 1.19a

Proportions. T0, T1, T2, and T3 indicate the mixture of red soil to RHA at volume ratios of 1:0, 1:1, 2:1, and 1:2, respectively. The same as below.

**Table 3:** Chemical properties of the mixture of rice husk ash and red soil with various proportions.

Treatment	pH	Available P (mg kg^−1^)	Available K (mg kg^−1^)	Organic matter (g kg^−1^)	Total N (%)	Ammonium nitrogen (mg kg^−1^)	Nitrate nitrogen (mg kg^−1^)
T0	4.64 ± 0.20b	5.58 ± 0.64d	100.00 ± 0.00d	6.85 ± 0.35d	0.05 ± 0.00b	18.90 ± 0.99a	25.90 ± 0.14b
T1	5.43 ± 0.28a	29.90 ± 4.67b	310.50 ± 9.19b	36.50 ± 0.42b	0.07 ± 0.00a	22.80 ± 0.57a	30.45 ± 1.34ab
T2	5.34 ± 0.06a	17.90 ± 1.27c	221.00 ± 26.87c	23.10 ± 5.52c	0.07 ± 0.01ab	21.25 ± 2.76a	27.75 ± 1.20ab
T3	5.66 ± 0.04a	59.10 ± 5.80a	406.00 ± 0.00a	58.95 ± 5.73a	0.09 ± 0.01a	23.35 ± 4.17a	31.70 ± 3.54a

### Seedling growth parameters

Plant height, stem diameter, seedling index and G value were enhanced by RHA application treatments, among which T1 performed the best, but no difference was observed in the emergence rate and root to shoot ratio between the RHA application and the control (Table 4). Similarly, the RHA addition also increased shoot dry (fresh) weight, root dry (fresh) weight and total dry (fresh) weight, and T1 documented the maximums among various treatments with the shoot, root and total dry weight being greater by 1.3, 1.0 and 1.2 times than the control, respectively (Table 5). Root architectures were improved by the RHA application (Table 6). Total root length, root surface area and numbers of tips were greater in T1 and T2, and root volume was greater in T1 relative to T0. No difference was observed in these root parameters between T2 and T0 (Table 6). The above results mean only a higher RHA application rate ≥ 50% volume ratio can contribute to the occurrence and growth of roots. T1 was found to be the optimal treatment with the best root architecture properties.

**Table 4:** Effects of the mixture of rice husk ash and red soil with various proportions on growth parameters of cotton seedlings.

Treatment	Emergence rate (%)	Plant height (cm)	Stem diameter (mm)	Root to shoot ratio	Seedling index	G value (mg day^−1^)
T0	0.94 ± 0.03a	16.42 ± 1.29c	1.90 ± 0.09b	0.13 ± 0.04a	0.57 ± 0.11c	0.07 ± 0.00d
T1	0.96 ± 0.03a	25.77 ± 2.64a	2.36 ± 0.13a	0.12 ± 0.02a	1.07 ± 0.07a	0.16 ± 0.02a
T2	0.95 ± 0.02a	19.96 ± 0.94b	2.06 ± 0.11b	0.14 ± 0.02a	0.76 ± 0.03bc	0.10 ± 0.01b
T3	0.95 ± 0.03a	26.08 ± 1.50a	2.31 ± 0.02a	0.13 ± 0.05a	0.93 ± 0.22ab	0.13 ± 0.00c

**Table 5:** Effects of the mixture of rice husk ash and red soil with various proportions on fresh (dry) weight of cotton seedlings.

Treatment	Shoot fresh weight (g)	Shoot dry weight (g)	Root fresh weight (g)	Root dry weight (g)	Total fresh weight (g)	Total dry weight (g)
T0	2.47 ± 0.17c	0.67 ± 0.03d	0.75 ± 0.24b	0.09 ± 0.02b	3.21 ± 0.09	0.76 ± 0.02d
T1	5.28 ± 0.52a	1.51 ± 0.18a	1.67 ± 0.14a	0.18 ± 0.02a	6.95 ± 0.52a	1.69 ± 0.18a
T2	3.43 ± 0.20b	0.90 ± 0.09b	1.17 ± 0.03ab	0.13 ± 0.00ab	4.60 ± 0.18c	1.03 ± 0.08c
T3	4.06 ± 0.36b	1.28 ± 0.07c	1.25 ± 0.57ab	0.16 ± 0.06a	5.31 ± 0.34b	1.44 ± 0.04b

**Table 6:** Effects of the mixture of rice husk ash and red soil with various proportions on root architectures of cotton seedling.

Treatment	Total length (mm)	Surface area (cm^2^)	Root volume (cm^3^)	Average diameter (mm)	Number of tips	Root vigor (μg g^−1^ h^−1^)
T0	124.43 ± 19.65b	23.64 ± 4.75b	0.36 ± 0.09b	0.60 ± 0.05ab	78.22 ± 22.69b	16.68 ± 0.61a
T1	245.90 ± 30.62a	52.76 ± 10.11a	0.92 ± 0.26a	0.68 ± 0.09a	186.33 ± 62.96a	20.47 ± 7.38a
T2	143.92 ± 26.03b	24.91 ± 4.25b	0.34 ± 0.06b	0.55 ± 0.01b	76.44 ± 33.48b	16.74 ± 7.27a
T3	223.15 ± 37.69a	39.77 ± 9.28a	0.57 ± 0.19b	0.56 ± 0.06b	167.89 ± 53.63a	19.16 ± 3.34a

### Seedling physiological parameters

**Figure 1:**
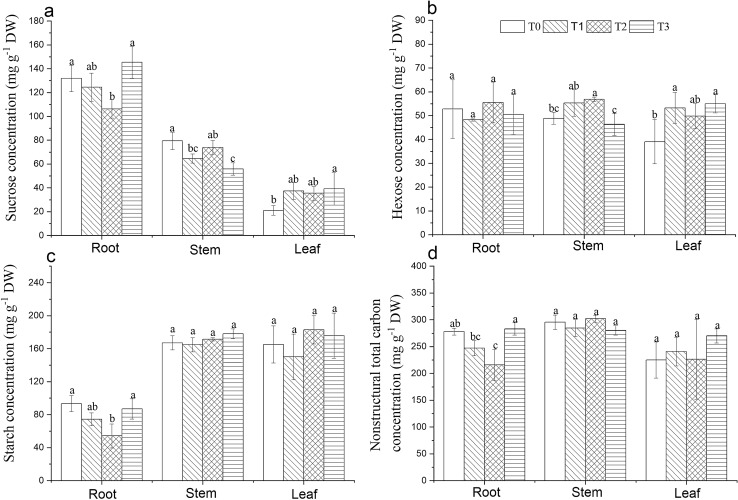
Effects of the mixtures of red soil and rice husk ash with various proportions on the sucrose (**a**), hexose (**b**), starch (**c**) and total nonstructural carbohydrate (**d**) concentrations in the root, stem and leaf of cotton seedlings. Histograms denote means ± SD, and diverse letters over which indicate significant difference at *P* ≤ 0.05 level. T0, T1, T2, and T3 represent the mixture of red soil to RHA at volume ratios of 1:0, 1:1, 2:1, and 1:2, respectively.

Sucrose and hexose concentrations in leaves were increased with the application of RHA, but no difference in the starch and total nonstructural carbohydrate concentrations (Fig. 1), which means the RHA application may improve the photosynthetic capacity of the cotton seedling leaf. Sucrose, starch and total nonstructural concentrations in roots were decreased with the application of RHA except T3, and T2 showed significant differences from T0 (Fig. 1a,c,d). Sucrose concentration in stems was decreased, but hexose concentration was increased by the RHA application (Fig. 1a,b). The application of RHA did not influence root vigor (Table 6). Total carbon concentration in the stem and root did not change with the application of RHA, but total nitrogen contents were decreased which led to enhanced carbon to nitrogen ratios in the stem and root (Table 7). The RHA application increased total carbon and total nitrogen accumulations summed across the whole plant with the maximums recorded in T1 (Table 7).

**Table 7:** Effects of the mixture of rice husk ash and red soil with various proportions on the carbon and nitrogen status in different parts of cotton seedlings.

Treatment	Total carbon concentration (g kg^−1^)	Total carbon per plant (mg)	Total nitrogen content (%)	Total nitrogen per plant (mg)	Carbon to nitrogen ratio
**Root**					
T0	437.72 ± 5.67a	39.34 ± 8.45b	1.44 ± 0.16a	1.32 ± 0.43a	30.61 ± 3.86c
T1	400.21 ± 19.14a	71.45 ± 8.77a	1.01 ± 0.03b	1.80 ± 0.21a	39.60 ± 0.62b
T2	406.78 ± 32.91a	51.90 ± 2.75b	1.06 ± 0.07b	1.36 ± 0.09a	38.26 ± 2.60b
T3	419.16 ± 25.15a	52.68 ± 8.32b	0.90 ± 0.13b	1.07 ± 0.11a	47.10 ± 3.97a
**Stem**					
T0	464.77 ± 1.76a	167.82 ± 6.92b	0.59 ± 0.01a	2.11 ± 0.10c	79.46 ± 0.86c
T1	462.85 ± 1.92a	419.22 ± 59.98a	0.44 ± 0.03c	3.96 ± 0.36a	105.61 ± 6.54a
T2	453.92 ± 17.75a	218.09 ± 17.07b	0.49 ± 0.01b	2.34 ± 0.08c	93.31 ± 4.67b
T3	459.36 ± 4.17a	383.74 ± 10.58a	0.41 ± 0.01d	3.39 ± 0.10b	113.30 ± 3.27a
**Leaf**					
T0	470.35 ± 4.49a	147.33 ± 7.58c	1.61 ± 0.21a	5.01 ± 0.34b	29.56 ± 3.37a
T1	473.77 ± 0.48a	285.83 ± 34.12a	1.42 ± 0.12a	8.52 ± 0.42a	33.51 ± 2.75a
T2	469.58 ± 11.41a	198.64 ± 36.28bc	1.31 ± 0.18a	5.45 ± 0.44b	36.40 ± 5.14a
T3	473.02 ± 11.57a	209.44 ± 23.07b	1.31 ± 0.17a	5.79 ± 1.04b	36.59 ± 4.44a
**Sum**					
T0	–	354.49 ± 7.57d	–	8.44 ± 0.68c	42.20 ± 3.90c
T1	–	776.50 ± 87.63a	–	14.28 ± 0.83a	54.29 ± 3.58b
T2	–	468.63 ± 53.80c	–	9.14 ± 0.41bc	51.21 ± 4.61b
T3	–	645.85 ± 28.05b	–	10.25 ± 1.06b	63.36 ± 5.64a

### Comparison of a synthetic growing media containing RHA and a commercial growing media in facilitating cotton seedling growth

In experiment 1, the mixture of red soil and RHA at a 1:1 volume ratio (T1) was convinced to be the optimal growing media for cotton seedlings in terms of its benefits on soil and seedling properties in combination with cost-efficiency. Further, we examined the effects of super absorbent polymers (SAP) at various application rates, and mepiquat chloride (MC) involved in different usage doses and application timings on cotton seedling growth on the T1 treatment basis. Two g L^−1^ SAP as potassium polyacrylamide and 50 mg L^−1^ MC foliar painting at the one-true-leaf stage were determined as the most suitable treatment following a series of gradient tests, respectively (data not shown). In experiment 2, a synthetic growing media involved in T1 combined with SAP was employed to examine its application perspective in nursing cotton seedling with a widely used commercial growing media (CM) as the control. A total of four treatments were included in experiment 2. Except T0, the other three treatments were subjected to the same MC foliar painting of 50 mg L^−1^ at the one-true-leaf stage. The usage of MC was aimed to oppress tall-footed seedlings and strengthen root architecture. Three treatments with MC application had greater stem diameter, root to shoot ratio, seedling index, root fresh weight, root dry weight and root vigor than that without MC application (Tables 8, 9, 10). Compared to CM + MC(T7), T1 + SAP + MC (T6) recorded greater shoot fresh weight and total fresh weight, but smaller plant height, root fresh weight and root dry weight (Tables 8, 9). In the case of root architectures, total root length and number of root tips were greater, but the average diameter was smaller in CM + MC than in T1 + SAP + MC. The other growth parameters and root characteristics did not differ from each other (Table 10). Notably, both treatments had much better performance than the pure soil treatment (T1) in improving seedling properties. Taken together, the growing media (RHA mixed with red soil in a volume ratio of 1:1 plus 2 g L^−1^ SAP) is comparable to the commercial media in nursing cotton seedlings.

**Table 8:** Effects of different growing media combined with MC application on growth parameters of cotton seedlings. *CM* commercial media, *SAP* super absorbent polymer, *MC* mepiquat chloride foliar painting at the one-true-leaf stage.

Treatment	Plant height (cm)	Stem diameter (mm)	Root to shoot ratio	Seedling index	G value (mg day^−1^)
T0	9.89 ± 0.49b	2.05 ± 0.03c	0.17 ± 0.02b	0.99 ± 0.06c	0.07 ± 0.01b
T1 + MC	8.06 ± 0.40c	2.25 ± 0.08b	0.27 ± 0.02a	1.26 ± 0.13b	0.06 ± 0.00b
T1 + SAP + MC	9.72 ± 0.27b	2.76 ± 0.04a	0.23 ± 0.04a	2.33 ± 0.12a	0.12 ± 0.01a
CM + MC	10.91 ± 0.22a	2.77 ± 0.04a	0.23 ± 0.01a	2.46 ± 0.05a	0.13 ± 0.00a

**Table 9:** Effects of different growing media combined with MC application on the fresh (dry) weight of cotton seedlings. *CM* commercial media, *SAP* super absorbent polymer, *MC* mepiquat chloride foliar painting at the one-true-leaf stage.

Treatment	Shoot fresh weight (g)	Shoot dry weight (g)	Root fresh weight (g)	Root dry weight (g)	Total fresh weight (g)	Total dry weight (g)
T0	3.69 ± 0.42c	0.74 ± 0.11b	1.31 ± 0.05d	0.13 ± 0.01d	5.00 ± 0.39c	0.87 ± 0.11b
T1 + MC	3.08 ± 0.11d	0.61 ± 0.04b	1.97 ± 0.22c	0.16 ± 0.01c	5.04 ± 0.18c	0.77 ± 0.04b
T1 + SAP + MC	7.83 ± 0.18a	1.26 ± 0.19a	3.68 ± 0.04b	0.28 ± 0.01b	11.50 ± 0.19a	1.53 ± 0.19a
CM + MC	6.12 ± 0.19b	1.37 ± 0.01a	4.14 ± 0.14a	0.32 ± 0.01a	10.26 ± 0.10b	1.69 ± 0.00a

**Table 10:** Effects of different growing media combined with MC application on the root architectures of cotton seedlings. *CM* commercial media, *SAP* super absorbent polymer, *MC* mepiquat chloride foliar painting at the one-true-leaf stage.

Treatment	Total length (mm)	Surface area (cm^2^)	Root volume (cm^3^)	Average diameter (mm)	Number of root tips	Root vigor (μg g^−1^ h^−1^)
T0	94.30 ± 1.47c	21.23 ± 1.13b	0.37 ± 0.01b	0.72 ± 0.04b	91.11 ± 5.67c	73.61 ± 3.09c
T1 + MC	110.21 ± 7.08c	25.23 ± 1.61b	0.47 ± 0.03b	0.74 ± 0.01b	95.00 ± 8.41c	84.23 ± 2.70b
T1 + SAP + MC	166.36 ± 14.95b	54.08 ± 6.44a	1.47 ± 0.39a	1.08 ± 0.16a	162.67 ± 17.78b	114.79 ± 4.53a
CM + MC	194.40 ± 12.85a	51.31 ± 3.59a	1.13 ± 0.27a	0.87 ± 0.14b	287.56 ± 8.59a	119.12 ± 6.76a

## Discussion

Rice husk is produced in large quantity during the process of rice milling which can be burned to generate steam or electricity, and the resultant rice husk ash (RHA) is left. It will be a pollutant on inappropriate disposal. RHA is characterized by lightweight, high SSA, porosity and durability, and good chemical stability^8,29^. Thus, it presents an enormous application value as a bioadsorbent for the removal of heavy metals and other toxic organic compounds from contaminated soil and water, but its utilization as a growing media is less investigated. In the present study, RHA incorporation into red soil at different rates was found to improve soil properties and cotton seedling growth parameters. RHA has a wide range of attributes similar to biochar like RHB^10,16^, but draw much less attention to its application in the agriculture industry than biochar, and thus relevant reports are limited. The following discussion is presented largely combined with biochar related studies.

Soil bulk density was decreased by the application rate of RHA equal to or above 50% by volume (Table 2). RHA incorporation rate at 33% by volume (T2) did not significantly modify the soil properties such as bulk density and total porosity (Table 2). The decreased bulk density in the mixed red soil should be derived from the small bulk density of RHA itself, a similar result as reported by Chang et al.^22^ who found decreased bulk density in sandy soil by pinewood biochar. Scanning electron micrograph (SEM) and transmission electron micrograph (TEM) analyses of the RHA microstructure indicated two kinds of pores present in RHA with one being microsized pores (around 10 μm) formed by interlacing of the fiber sheet and the other one being nanosized pores (< 50 nm) formed by nano SiO_2_ particles^30^. The nanopores and nano SiO_2_ particles are the crucial contributors to specific surface area and high activity of RHA. Similarly, Singh et al. also observed various macro- and micro-pores on the surface of RHA^14^. The existence of pores with a diameter range of 0.1 to 10 μm in a crop straw biochar can help improve the WHC of soil^31^. The biochar application rate was positively correlated with the WHC of biochar-amended soil when the former was below 20%, which means the presence of an optimal value of biochar application rate for maximizing the WHC^22^. The present study indicated a positive correlation between WHC and RHA application rate (Table 2).

The pH value was increased with increasing RHA incorporation rate (Table 3). RHA was moderately alkaline with a pH of 7.89 (Table 1) and featured by high neutralizing power, which is suitable for neutralization of acid soils^17^. The red soil in the present study is originally acidic and turned less acidic after the RHA incorporation (Table 3), as observed by Masulili et al.^12^. The higher pH of RHA may be attributed to the formation of carbonates during the combustion process^12^. Soil pH can affect the microbial abundance, diversity, and activity^32^. For example, the moderate alkalinity of RHA benefits soil bacterial communities accounting for rapid mineralization of organic matter and its microbial recruitment^33^. Typical acid red soil is a shortage of available K, P, Ca and Mg due to leaching under the subtropical monsoon climate with high precipitation and temperature^2^. By contrast, RHA is rich in available K and P (Table 1). Thus, RHA can directly enhance the nutrient availability of red soil as a primary source of nutrients. On the other hand, RHA can indirectly improve nutrient supply to plants owing to its high active surface, and the existence of couples of reactive functional groups affecting adsorption^17,34^. Fourier transform infrared spectroscopy (FTIR) analysis of RHA indicated it carries functional groups like hydroxyl (–OH), silanols (Si–OH) and siloxanes (Si–O–Si–OH) groups, but carboxylic radicals (–COOH) was not detected^10^. RHA is expected to increase soil CEC because of its good porosity and large and predominately negative charged surface functional groups, and the adsorption of organic matter on RHA surfaces. More cationic nutrients can be absorbed by a soil with a higher CEC than that with a lower CEC, so nutrients are retained other than leached and thus more available for plant uptake^35^.

RHA application improved the cotton seedling growth (Tables 4, 5), which is partly attributed to enhanced nutrient availability and pH (Table 3). Tian et al. reported that biochar derived from green waste combined with peat at a ratio of 1:1 by volume enhanced total biomass and leaf surface area of *Calathea rotundifolia* relative to peat substrate alone, which was associated with improved substrate attributes and increased nutrient availability on biochar addition^36^. Lettuce biomass was increased by 184–270% after the replacement of 10% peat (by volume) by sewage sludge biochar, which was attributed to enhanced N, P and K contents and microbial activities in growing media^37^. Increased biomass accumulation may be the result of increased inorganic nutrient uptake and photosynthetic production together. The former can be owing to enhanced nutrient availability due to RHA incorporation. The latter could be explained by the carbohydrate change in leaves occasioned by RHA addition. The hexose and sucrose concentrations in leaves were increased, but starch concentration was not affected by the RHA addition (Fig. 1a–c), suggesting greater photosynthetic productivity and carbon export availability in leaves. The root architecture of cotton seedlings was improved by RHA application treatments, among which T1 recorded the best performance (Table 4). Similar observations were reported in Muscadine grape (*Vitis rotundifolia* L.) and sugarcane^22,38^. Pinewood biochar addition to pure sandy soil improved soil physical characteristics and facilitated the root growth and development of potted Muscadine grape (*Vitis rotundifolia* L.)^22^. Cassava stem derived biochar application enhanced the soil fertility and pH, and improved the root properties of sugarcane seedlings in a potted experiment^38^. The increased root growth may be ascribable to improved soil physical properties helping the roots more easily access to air and water and less acidic pH which may be beneficial to microbial activities^33^. On the other hand, the improved root growth may be associated with the enhanced soluble sugars concentration in the leaf which is typically considered as an indicator of active photosynthesis under normal growth conditions^39^. For instance, the root growth of *Arabidopsis* seedlings at the early stage was prompted by the photosynthetic sucrose as a long-distance signal molecule^40^. Exogenous application of sugar can increase free auxin levels and basipetal auxin transport in *Arabidopsis* seedlings and promote the elongation of the hypocotyl and roots^41^. Sucrose application to the shoot of *Arabidopsis* alone triggered the emergence of lateral root primordia^42^. Druege et al. found that increased rooting of chrysanthemum cuttings was associated with higher sucrose: starch ratios in leaves^43^. The result accords well with the present study where increased sucrose concentration in combination with an invariable starch concentration in leaves due to RHA incorporation produced increased sucrose to starch ratios which were accompanied by enhanced root tips (Table 6, Fig. 1b,c).

T1 + SAP + MC and CM + MC performed better than the two other treatments, and did not differ significantly from each other in a majority of parameters tested (Tables 8, 9, 10). Peat is a major composition included in commercial growing media pushing its price to a high level, which is a non-renewable source due to its very long regeneration time. A large amount of exploitation of peat will impose severe damage on the environment as it provides certain ecosystem services such as biodiversity, water regulation and C sinks^17^. RHA possesses many physico-chemical properties similar to peat, and its usage as an additive to red soil exhibited desirable effects on nursing seedlings comparable to commercial growing media. Thus, RHA will be a promising substitute for peat as growing media, which is also of great implication for the development of circular economy in the agriculture sector.

## Conclusions

Red soil is characterized by low pH value, and deficient available P and K, total N, and organic matter. The addition of RHA counteracts the acidity of red soil and improves the nutrient availability to plants. Bulk density was decreased and water holding capacity and total porosity were increased in red soil with increasing application rate of RHA. The RHA incorporated soils favored the growth of cotton seedlings with improved shoot morphological traits and root architectures. The application rate at a volume ratio of 1:1 of RHA to red soil was found to be optimal for growing cotton seedlings in the present study. The mixture of RHA and red soil at a 1:1 volume ratio plus 2 g L^−1^ SAP exhibited a high nursing seedling efficiency comparable to a commercial growing media under the condition of foliar application of mepiquat chloride at the one-true-leaf stage. RHA is a desirable substance for ameliorating the red soil properties and can replace peat as growing media for nursing cotton seedlings.

## Data Availability

Raw data were generated at College of Agronomy Jiangxi Agricultural University. Derived data supporting the finding of this study are available from the corresponding author (FT) on request.
